# Dataset for understanding the effort and performance of external auditors during the COVID-19 crisis: A remote audit analysis

**DOI:** 10.1016/j.dib.2022.108119

**Published:** 2022-04-01

**Authors:** Saeed Rabea Baatwah, Ali Ali Al-Ansi

**Affiliations:** a Shaqra University, Saudi Arabia; b Seiyun University, Yemen; c The Central Organization for Control and Auditing, Yemen

**Keywords:** COVID-19 pandemic, Remote audit, Audit effort, Auditor performance, Saudi Arabia, Audit firms

## Abstract

The onset of the COVID-19 pandemic at the end of 2019 has dramatically changed the life of both individuals and corporate businesses. Besides the health fears of the pandemic, there are sizable challenges for some jobs that habitually deliver their services face-to-face, disrupting the quality of service. In particular, external auditors, the most critical and reliable accreditors of financial reports, are in an unprecedented situation to conduct audit works and collect sufficient and appropriate audit evidence. To capture how COVID-19 affects auditors in Saudi Arabia, an online questionnaire was developed and distributed from April to August 2021 via email to all 417 auditors/audit firms registered with the Saudi Organization for Chartered and Professional Accountants (SOCPA). This dataset contains responses from 193 auditors, in four major sections: demographic information (7 items); audit effort during COVID-19 (3 items); auditor performance during COVID-19 (7 items); and remote audit proficiency (6 items). It may serve for theoretical and practical assessment for auditors in the time of COVID-19, for example, evaluating how auditors conduct their work during the lockdown and how remote audit proficiency impacts the effort and performance of auditors. In addition, it can be used to assess the differences between auditors by considering the moderating effect of their personal characteristics on the impact of remote audit proficiency on both effort and performance.

## Specifications Table

**Table:** 

Subject	Economics, Econometrics and Finance
Specific subject area	Accounting and Taxation
Type of data	Primary data, tables, and questionnaire in PDF file
How data were acquired	Data were gathered using online-questionnaire (Google Form)
Data format	Raw data Filtered
Parameters for data collection	The data were obtained from all registered audit firms in Saudi Arabia; a list of 417 auditors was identified and selected as the population of this study.
Description of data collection	To collect the data, the online questionnaire was emailed to each audit firm, requesting them to distribute it among their auditors.
Data source location	City/Town/Region: All cities: Riyadh, Jeddah, Al Dammam, Almadinah, etc. Country: Saudi Arabia
Data accessibility	Repository Name: Mendeley Data Data identification number: 10.17632/3d57m4n6g5.1 Direct link to dataset: https://data.mendeley.com/datasets/3d57m4n6g5/1

## Value of the Data

• This dataset provides an interesting dataset assessing the work of external auditors in terms of effort, performance, and the use of technology. The value of this dataset lies in examining how auditors react in the recent COVID-19 crisis and how they can supply high-quality audit as there is a doubt on the ability of auditors to ensure the quality of their works during the pandemic.
• The dataset will be useful for those who are interested in studying the performance and effort of auditors during COVID-19.
• The dataset will be beneficial for researchers and professionals interested in researching the remote audit proficiency and its impact on the effort and performance of auditors during the pandemic.
• The dataset can be used to analyze how the relationship between remote audit proficiency and both effort and performance are indirectly affected by personal characteristics of auditors.
• The data were collected for a sample of professional external auditors in Saudi Arabia and therefore represent a unique dataset for an underexplored setting where several socio-economic and cultural factors are unavailable in several audit markets.

## 1 Data Description

Auditors in normal times conduct most of their work on their clients’ site, but during COVID-19 it became impossible to physically attend their clients [1]. Thus, auditors during COVID-19 were put in the unprecedented situation of ensuring that they could continue to deliver high-quality audit [2]. This dataset provides information on remote audit proficiency, audit effort, and audit performance during the pandemic. This information, stored as raw data in a Microsoft Excel Worksheet (xls), was obtained by online questionnaire from 193 external auditors from April to August 2021, in the Kingdom of Saudi Arabia.^1^ The returned responses were all usable and represented 46% of the whole auditors’ population in Saudi Arabia. The dataset comprises four major sections detailing items related to auditors’ demographic information (7 items), audit effort (3 items), auditor performance (7 items), and remote audit proficiency (6 items). The respondents rated the items of interest using a five-point Likert scale (e.g., 1 for Strongly disagree and 5 for Strongly agree). The questionnaire was provided as a supplementary file. We include all items on the questionnaire in Table 1, Table 2, Table 3, Table 4. Table 1 presents the primary description of demographic characteristics. Table 2, Table 3, Table 4 provide detailed assessments of the responses in relation to audit effort, auditor performance, and remote audit proficiency.

**Table 1:** Respondents’ characteristics.

Variable	Categories	Freq.	%
Position			
	Audit trainee	25	13.0
	Auditor	39	20.2
	Senior auditor	30	15.5
	Audit manager	36	18.7
	Partner	63	32.0
Age			
	20 – 29	54	28.0
	30 – 39	57	29.5
	40 – 49	40	20.7
	50 and above	42	21.8
Education			
	Diploma	2	1.0
	Bachelor	124	64.2
	Master	53	27.5
	Doctoral	6	3.1
	Other	8	4.2
Major			
	Accounting	188	97.4
	Others	5	2.6
Experience (years)			
	1-5	76	39.4
	6-10	63	32.6
	11-15	20	10.4
	16- more	63	32.6
Participate in audit during COVID-19			
	YES	169	87.6
	NO	24	12.4
Which accounting period ended participated			
	2019	26	13.5
	2020	14	7.3
	Both	133	68.9
	No participate	20	10.4

**Table 2:** Items for audit effort (EFORT).

No	Items	Response										
Rate your audit efforts during COVID19 pandemic with your audit efforts prior to COVID19 pandemic (1=Much less and 5=Much higher)		Much less	Less	Similar	Higher	Much higher	Mean	S.D.	Loading	α^a^	CR^b^	AVE^c^
1	How do you rate yourself in terms of the quantity of audit work you accomplish?	2.6	17.1	19.7	46.1	14.5	3.5	1.0	0.841	0.759	0.863	0.679
2	How do you rate yourself in terms of your ability to reach your planned audit activities?	1.6	18.1	26.9	42.0	11.4	3.4	1.0	0.910			
3	How do you rate yourself in terms of the evaluation you have received from your audit manager(s)/partner(s)?	1.6	16.1	35.2	40.9	6.2	3.3	1.0	0.708			

a α = Cronbach's alpha.

b CR = Composite Reliability.

c AVE = Average Variance Extracted.

**Table 3:** Items for auditor's performance (PERF).

No	Items	Response										
Audit quality/performance (Rate your audit quality or performance during COVID19 pandemic with your audit quality or performance prior to COVID19 pandemic (1=Much less and 5 Much higher)		Much less	Less	Similar	Higher	Much higher	Mean	S.D.	Loading	α^a^	CR^b^	AVE^c^
1	How do you rate yourself in terms of the quantity of audit work you accomplish?	2.1	15.5	44.6	32.6	5.2	3.2	0.9	0.645	0.803	0.858	0.503
2	How do you rate yourself in terms of your ability to reach your planned audit activities?	0.5	10.9	45.6	34.2	8.8	3.4	0.8	0.790			
3	How do you rate yourself in terms of the evaluation you have received from your audit manager(s)/partner(s)?	1.6	4.7	48.7	34.7	10.4	3.5	0.8	0.653			
4	How do you rate yourself in terms of the quality of your audit skepticism and objectivity?	1.0	14.0	34.2	41.5	9.3	3.4	0.9	0.698			
5	How do you rate yourself in terms of your ability to manage time and expenses?	1.0	25.4	29.5	36.3	7.8	3.2	1.0	0.686			
6	How do you rate yourself in terms of the respect you have received from others for your audit performance?	0.5	10.4	49.2	30.1	9.8	3.4	0.8	0.771			
7	How do you rate yourself in terms of the quality of your performance with regard to the use of appropriate audit procedures in the appropriate circumstances?	1.0	8.3	44.6	37.3	8.8	3.5	0.8	0.645			

a α = Cronbach's alpha.

b CR = Composite Reliability.

c AVE = Average Variance Extracted.

**Table 4:** Items for remote audit proficiency (RMOTA).

No	Items	Response										
Rate your skills and abilities to use information and communication technology in conducting audit activities (1=Rarely and 5=Always)		Rarely	Sometimes	Often	Usually	Always	Mean	S.D.	Loading	α^a^	CR^b^	c^c^
1	I use email, telephone and/or web conference to arrange engagement procurement.	6.7	20.7	19.7	31.1	21.8	3.4	1.2	0.653	0.857	0.893	0.587
2	I use virtual teams, web conference and/or electronic workpaper system to discuss and assign audit planning.	8.3	17.1	29.0	29.5	16.1	3.3	1.2	0.784			
3	I use videoconferencing, connect to the client system over the network, run analytical tests through a terminal, and/or check audit logs to evaluate internal control and compliance.	11.9	26.4	22.8	20.7	18.1	2.8	1.4	0.862			
4	I use client system over the network and closed-circuit video to conduct substantive testing.	24.9	30.6	20.2	17.6	6.7	2.5	1.2	0.832			
5	I use web conferencing with employees in charge, management, and audit committees for audit decisions and reporting.	15.0	20.2	25.9	27.5	11.4	3.0	1.2	0.842			
6	I train myself with more related information and communication technology systems such enterprise resource planning system, computer-assisted auditing techniques, and/or other electronics techniques used in audit.	13.5	12.4	25.4	27.5	21.2	3.3	1.3	0.580			

a α = Cronbach's alpha;

b CR = Composite Reliability;

c AVE = Average Variance Extracted.

## 2 Experimental Design, Materials and Methods

This dataset contains responses from 193 auditors in Saudi Arabia obtained within the timeframe April to August 2021. A descriptive online survey design was adopted to evaluate auditors’ perception of their effort and performance during COVID-19, and how remote audit has been embedded as a normal practice in their business model. The questionnaire was originally written in English and shared with audit professors and audit partners to assess the face reliability and readability of the content. After the suggested amendments had been made, it was translated into Arabic language by experts. This online survey (questionnaire) was emailed as a Google Form link to all registered audit firms in Saudi Arabia (25 audit offices/firms), based on the email address provided on the website of SOCPA. According to the list of firms available on the website in early April, 417 audit partners and staff were associated with these firms. Thus, a friendly appreciative request was inserted in the email requesting the audit firm to share the link with all auditors in the firm.

The questionnaire included four sections developed from an extensive review of research on the considered constructs. For example, the first section describes the demographic characteristics of the respondents, including position within the firm, age, education, experience, involvement in auditing during the pandemic, and the year of the involvement. The second section presents three items for assessing the effort of auditors (EFORT) during COVID-19. This section was adapted from prior research [4] and requested the respondents to compare their effort before and during COVID-19, based on a five-point Likert scale (from 1= much less to 5= much higher). Table 2 details these items and their primary statistics. The third section contains seven items examining how auditors perceive their performance (PERF) of major audit activities during COVID-19. These items were based, with some modification, on prior research [5,6]; the auditors were asked to rate their performance during COVID-19 in comparison with performance prior to the pandemic, using a five-point Likert scale (from 1= much less to 5 = much higher). Table 3 presents these items with primary results. The final section lists six items exploring the auditors’ experience with remote audit (RMOTA). These items were developed based on the analytical framework suggested by prior research [7,1] on remote audit. They evaluate the auditor's use of more advanced technology in conducting financial statements audit, measured on a five-point Likert scale (from 1 = rarely to 5 = always). Table 4 shows these items and their associated major statistical description.

## Ethics Statement

This study does not involve the use of human subjects, animal experiments or data collected from social media platforms. Furthermore, in the country where the study was conducted, ethics approval from an appropriate local ethics committee is not required prior to conducting the study. Nonetheless, the authors obtained the informed consent of all participants, who were also informed in the preface to the questionnaire that their participation in the study was totally voluntary, and that anonymity and confidentiality of their personal information would be strictly adhered to. Thus, the authors guarantee in this dataset that personal data, such as name, ID number and emails are anonymized.

## CRediT authorship contribution statement

**Saeed Rabea Baatwah:** Methodology, Data curation, Writing – original draft, Investigation. **Ali Ali Al-Ansi:** Methodology, Data curation, Writing – original draft, Writing – review & editing.

## Declaration of Competing Interest

The authors declare that they have no known competing financial interests or personal relationships that could have appeared to influence the work reported in this paper.

## Data Availability

Dataset for effort, performance & remote audit: A survey in COVID-19 (Original data) (Mendeley Data).

## Appendix Supplementary materials

Image, application 1

Image, application 2

## Footnotes

1 We verified the non-response bias using procedures suggested by prior research [3]. Comparing the first month's responses with the last month's, two-tailed t-test results, untabulated, revealed no significant differences between the groups, indicating no bias.

## References

[1] Christ M.H., Eulerich M., Krane R., Wood D.A. (2021). New frontiers for internal audit research. Account. Perspect..

[2] Appelbaum D., Budnik S., Vasarhelyi M. (2020). Auditing and accounting during and after the COVID-19 crisis. CPA J..

[3] Armstrong J.S., Overton T.S. (1977). Estimating nonresponse bias in mail surveys. J. Market. Res..

[4] Krishnan B.C., Netemeyer R.G., Boles J.S. (2002). Self-efficacy, competitiveness, and effort as antecedents of salesperson performance. J. Pers. Sell. Sales Manag..

[5] Fogarty T.J., Kalbers L.P., Arnold V., Clinton B.D., Luckett P., Roberts R., Wolfe C., Wright S. (2006).

[6] Kalbers L.P., Cenker W.J. (2008). The impact of exercised responsibility, experience, autonomy, and role ambiguity on job performance in public accounting. J. Manag. Issues.

[7] Teeter R.A., Alles M.G., Vasarhelyi M.A. (2010). The remote audit. J. Emerg. Technol. Account..

