# Supplementary material for: Dataset for understanding the effort and performance of external auditors during the COVID-19 crisis: A remote audit analysis
Source: Data Brief. 2022 Apr 1;42:108119. doi: 10.1016/j.dib.2022.108119 (PMC8971104; doi:10.1016/j.dib.2022.108119)
Supplement: Supplementary file 1 [file mmc1.pdf]

The status of Saudi auditors in COVID-19 pandemic

We are currently conducting a research assessing the influence of COVID19 pandemic on the audit practices in Saudi Arabia. In this regard, we would like to invite you to be a respondent to this survey. Your valuable contribution will provide useful inputs as it would help to achieve the objectives of this study. We would like to notify you that responding to the items in the questionnaire should be based on your actual practice not on assumed practice. Please be assured that all information provided will be kept strictly confidential as the finding will be presented on an aggregate basis to be used solely for academic purpose.

Please contact the researchers for any enquiry about this research, and thank you for your time and cooperation.

Yours faithfully,

Corresponding researcher:

Dr. Saeed Rabea Baatwah
College of Business Administration
Shaqra University
Email: sbaatwah@yahoo.com

<u>Questionnaire</u>

The status of Saudi auditors in COVID-19 pandemic

Section A: General Information
This section asks general questions match your general information.

Please tick the relevant number/statement that closely to your chosen answer or fill in the blank as appropriate:

1. My position within the organization

☐ **Partner** ☐ **Audit Manager** ☐ **Senior Auditor**
☐ **Auditor** ☐ **Junior Auditor**

2. Please tick your age range:

☐ **20 – 29** ☐ **30 – 39** ☐ **40 – 49** ☐ **50 and above**

3. Please tick your highest education.

☐ **Diploma** ☐ **Bachelor Degree** ☐ **Master Degree** ☐ **Doctoral Degree** ☐ **Other (please specify) ____________**

4. Please tick your major ☐ **Accountancy** ☐ **Other (please specify) __2__________**

5. Please tick your years' experience in auditing range:

☐ **1 – 5** ☐ **6 – 10** ☐ **1 – 15** ☐ **16 and above**

6- Have you conducted financial statements audit during COVID19 pandemic?

☐ **Yes** ☐ **No**

7- If you have conducted financial statements audit during COVID19, please specify in which accounting yearend?

☐ **2019** ☐ **2020** ☐ **Both** ☐ **No participate**

SECTION B: Variables of the study

Part Two: Audit efforts: (Rate your audit efforts during COVID19 pandemic with your audit efforts prior to COVID19 pandemic): Please tick the relevant number that closely to your chosen answer.

1= Much less	2= Less	3= Similar	4= Higher	5= Much higher

8.	Compared to a year prior to COVID19, the overall effort I exert into the audit activities is……	1	2	3	4	5
9.	Compared to a year prior to COVID19, the number of hours I spent in audit activities is ……	1	2	3	4	5
10.	Compared to a year prior to COVID19, the number of audit tests is…..	1	2	3	4	5

Part Three: Audit quality/performance (Rate your audit quality or performance during COVID19 pandemic with your audit quality or performance prior to COVID19 pandemic): Please tick the relevant number that closely to your chosen answer.

1= Much less	2= Less	3= Similar	4= Higher	5= Much higher

11.	How do you rate yourself in terms of the quantity of audit work you accomplish?	1	2	3	4	5
12.	How do you rate yourself in terms of your ability to reach your planned audit activities?	1	2	3	4	5
13.	How do you rate yourself in terms of the evaluation you have received from your audit manager(s)/partner(s)?	1	2	3	4	5
14.	How do you rate yourself in terms of the quality of your audit skepticism and objectivity?	1	2	3	4	5
15.	How do you rate yourself in terms of your ability to manage time and expenses?	1	2	3	4	5
16.	How do you rate yourself in terms of the respect you have received from others for your audit performance?	1	2	3	4	5
17.	How do you rate yourself in terms of the quality of your performance with regard to the use of appropriate audit procedures in the appropriate circumstances?	1	2	3	4	5

Part Four: Remote audit proficiency (Rate your skills and abilities to use information and communication technology in conducting audit activities): Please tick the relevant number that closely to your chosen answer.

1= Rarely	2= Sometimes	3= Often	4= Usually	5= Always

18.	I use email, telephone and/or web conference to arrange engagement procurement.	1	2	3	4	5
19.	I use virtual teams, web conference and/or electronic workpaper system to discuss and assign audit planning.	1	2	3	4	5

20.	I use videoconferencing, connect to the client system over the network, run analytical tests through a terminal, and/or check audit logs to evaluate internal control and compliance.	1	2	3	4	5
21.	I use client system over the network and closed-circuit video to conduct substantive tests.	1	2	3	4	5
22.	I use web conferencing with employees in charge, management, and audit committees for audit decisions and reporting.	1	2	3	4	5
23.	I train myself with more related information and communication technology systems such enterprise resource planning system, computer-assisted auditing techniques, and/or other electronics techniques used in audit.	1	2	3	4	5

Thank you very much for your cooperation
