# Supplementary material for: Dataset for understanding the effort and performance of external auditors during the COVID-19 crisis: A remote audit analysis
Source: Data Brief. 2022 Apr 1;42:108119. doi: 10.1016/j.dib.2022.108119 (PMC8971104; doi:10.1016/j.dib.2022.108119)
Supplement: Supplementary file 2 [file mmc2.pdf]

Pusstronics Partners

Gillian Martin & Alistair Sutcliffe

+44 (0)1625-583605
gillianalderley@yahoo.co.uk

3 Marlborough Avenue
Alderley Edge
Cheshire SK9 7HS, UK

18 March 2022

Dear Editor

This is to certify that the document submitted by Dr Saeed Rabea Baatwah, entitled *Dataset for understanding the effort and performance of external auditors during the COVID-19 crisis: A remote audit analysis* was proof read by a professional native English speaker, a partner in Pusstronics Partners.

Yours faithfully

G. Martin

Gillian Martin

Ms Gillian Martin (MA, MSc, CertEd, DipLib) has a background in information science and the teaching of computer applications, and for many years has been involved in the preparation for publication of reports, journal articles and text books. She has edited manuscripts in a variety of technical, scientific and humanities disciplines. Pusstronics Partners *was founded with Professor Alistair Sutcliffe in 2002.*
